# 

**Published:** 1910-08-20

**Authors:** 


### OEYNHAUSEN SPA.

To English medical men many Continental watering-places and bathing resorts are much too slightly known. One of these attractive spots that has suffered (so far as an English connection is concerned) through our ignorance of its virtues is Oeynhausen in Westphalia, one of the pleasantest and most charming hydropathic villages one can choose for a cure on the Continent. Oeynhausen, which dates back to the early 'thirties of the last century, is thoroughly up to date, and we may draw attention, among other things, to one of its features which our English spas may usefully imitate—the fine orthopædic institute that exists in connection with the baths. Visitors will find themselves well cared and catered for, at a charge which is reasonable, while paying hospital patients are received in the Johanniterasyl at a charge of less than 1s. 6d. a day. The Oeynhausen springs produce strong thermal $CO_2$ containing water, and is specially suited for joint and vascular cases. The Königliche Bæde Verwaltung—the London offices of which are at 23 Old Jewry, E.C.—issues an admirable descriptive booklet, beautifully illustrated, which tells the intending spa-pilgrim all about Oeynhausen for the moderate charge of 1s., while a smaller illustrated pamphlet can be obtained free on application to the London offices.

BOOKS RECEIVED.

Adlard and Co.
"Spa Treatment." By Dr. N. Wood.

The Oxford Medical Publications.
"Practical Nursing for Male Nurses in the R.A.M.C." By E. M. and A. R. Hassard.

Funk and Wagnalls Co.
"Story of the Locomotive." Supplements.

Longmans and Co.
"State and the Doctor." By S. and B. Webb.
"Principles of Gynæcology." By W. Blair Bell.

### Appointments and Resignations.

Dr. F. P. Piper has been elected by the Whitstable-on-Sea Urban District Council as medical officer in the place of Dr. J. W. Hayward (resigned).

Dr. Leonard Williams, M.D., M.R.C.P., has been appointed physician, Mr. Wallace Ashdowne, F.R.C.S., surgeon in charge of the nose, throat, and ear department, and Mr. Alexander Gow, M.B., B.S., pathologist and registrar, to the Miller General Hospital for South-East London.

In succession to Mr. E. C. C. Baly who has been appointed Professor of Chemistry in the University of Liverpool, Dr. R. H. Aders-Plimmer and Dr. W. B. Tuck have been appointed teachers of chemistry to medical students at University College. Mr. H. J. Page has been appointed demonstrator in chemistry and physiological chemistry to medical students.

### THE QUESTION BOX.

**SPECIAL NOTICE.**—Questions of interest affecting the honorary medical staff and hospital administration in all departments are invited. When any point arises we hope our readers will formulate it in a question and so co-operate to make this column of real value and interest. In this way the experience of the best-managed hospitals should be made helpful to the whole hospital world. We shall welcome the contribution of both questions and answers.

N.B.—The publication of an answer in this column does not necessarily preclude the publication of subsequent answers to the same question, for a question may involve points which require to be threshed out and fully discussed. Answers, if published, will be paid for at scale rates.

### HOSPITAL DISPENSING.

A Correspondent writes: The Committee of a hospital appoints a firm of chemists (duly qualified) on certain terms to dispense at the institution. The firm sends down an unqualified, but experienced, assistant to do the work. In the event of any misadventure or accident happening, who would be responsible? Is the Committee justified in continuing the arrangements; the representative of the firm gives satisfaction?

**Answer.**

If it was part of the agreement that the dispensing should be done by a qualified person there has been a breach of contract. There is, however, nothing in the Pharmacy Acts to require that dispensing in institutions of this kind should be done by qualified persons, and the responsible party would be the same, whether the dispenser were qualified or unqualified. Nevertheless, should an accident occur, the hospital authorities would not be liable to the same amount of moral blame if the dispenser were qualified, as they might be if the dispenser were unqualified. The point is clearly covered by the recent decision of Mr. Justice Walton in the case of Evans *v.* Liverpool Corporation, and another decision of the Court of Appeal in Hillyer *v.* St. Bartholomew's Hospital. The effect of those two cases is that hospital authorities are not responsible for the negligence of the nurses and doctors whom they employ, provided they have exercised reasonable care in selecting and appointing competent persons. The same principle applies to the case of a chemist. If he is a registered pharmaceutical chemist or chemist and druggist, it seems that the hospital authorities have done their duty to the public when they appointed him.

Low's Handbook to the Charities of London, 1910.—For three-quarters of a century this admirable little shilling reference manual has made its appearance with commendable regularity every year, and this time, too, it is prompt in its appearance and accurate in its information. The various institutions that are given are dealt with sufficiently broadly to make the information about them of real value to the searcher or inquiring student. So far as we have been able to judge there are no mistakes of commission in the book, and considering the small price that is asked for it, the little volume is a marvellous compilation. We would, however, suggest to the editor and publishers that future editions might contain some information with regard to various social charities—boys' and girls' clubs—which are at present not included in the book. The handbook can be procured from the publishers, Eliot Boothroyd, Leighton House, 168 Fleet Street, W.C.

### Gifts and Bequests.

The Grocers' Company has made a donation of £100 to St. Mary's Hospital, Paddington.

Mr. Robert Brown, of Laurel Bank, Holywood, Co. Down, bequeathed £100 to the Royal Victoria Hospital.

Mr. Henry Heymann, of Greencroft Gardens, South Hampstead, a director of Messrs. Metzler and Co., Limited, left £100 to the Jewish Hospital.

The Committee of the Poplar Hospital for Accidents has received £100 from an unknown donor, "A friend, per J. D. L."

Miss Elizabeth Ann Brown, of Ben Ledi, Buckhurst Road, Bexhill-on-Sea, bequeathed the residue of her estate, about £8,000, for charitable purposes.

The following donations have been received by the Middlesex Hospital: The Royal Warrant Holders' Association, £105; Messrs. Coutts & Co., £100.

Mr. Thomas Henry Holmes, formerly in business in Aldermanbury, Bradford, left £500 to the Bradford Royal Infirmary, and £100 to the Amblers Home, Hest Bank, Lancs.

The Mercers' Company has granted £21 in response to the appeal for £1,000 to mark the completion of twenty-one years' work of the Seaside Camps for London Working Boys.

Mr. John Heelas, of Whiteknights Park, Earley, Reading, head of Messrs. Heelas, Sons & Co. (Limited), drapers and house furnishers, bequeathed £100 to the Royal Berkshire Hospital.

Mr. Joseph Higginson, of Westbourne Road, Birkdale, Southport, a director of the Fine Cotton Spinners' and Doublers' Association, has directed that all his subscriptions to religious and charitable purposes should be continued for a year after his death.

Mrs. Lorina Hannah Liddell, of Ascot Wood, Ascot, President of the Committee of the Acland Nursing Home, Oxford, and later of the Nursing Home at Ascot, who died on June 25, aged 84, widow of the late Dean Liddell, left £100 each to the Sarah Acland Memorial District Nursing Fund, Oxford, and the Victoria Cottage Nursing Home, Ascot.

Miss Caroline Muston Cahill, of Avenue House, Richmond, Surrey, who died on June 7, aged 89, bequeathed £400 to the King's Daughters' Beds in Bethnal Green Mission Hospital, £200 to the Children's Convalescent Home, Brighton, £500 to the Richmond Hospital, and £250 to the Richmond Dispensary.

Mr. Arthur Lloyd, of Warren Hill, Washington, Pulborough, Sussex, a director of Messrs. Edward Lloyd, Limited, proprietors of the *Daily Chronicle* and of *Lloyd's Weekly News*, who left estate of which the net personalty amounts to £194,129, bequeathed £900 to be applied for carrying on the Convalescent Home at Rustington, or work of a similar character, and after a few other bequests left the residue of his property, which will apparently amount to at least £120,000, upon trust to apply the same for such one or more of the following charitable purposes, among others, as his trustee shall think fit: The purchase or the contribution towards the purchase of any open space or open spaces and recreation grounds, or the contribution to the funds of any associations, societies, or committees which provide public gardens, parks, or playing-fields; donations or contributions to the building or enlargement fund of any hospital, or the general funds of any such hospital or hospitals, or to the building fund of new hospitals or convalescent homes.

### Jottings.

Lady Hardinge of Penshurst has accepted the office of vice-patron of the Chelsea Hospital for Women.

The United Friendly Societies at Tallangatta, a township in rough country near the headwaters of the Murray, have handed over to a committee the sum of £280 for a proposed cottage hospital on a well-defined plan.

A very small settlement, Gunbower, on the Murray below Echuca, having had two most successful Hospital Sundays, is starting a cottage hospital to be worked in conjunction with the Bush Nursing Association.

A representative meeting of Lancastrians last Tuesday decided to raise public subscriptions in the borough and district to endow in perpetuity one or more beds in the Royal Lancaster Infirmary as a memorial to King Edward. The present King opened the institution fourteen years ago.

At a large meeting held at Lahore in connection with the King Edward memorial movement, it was proposed to build a new medical college at Lahore and to enlarge the existing hospitals so as to form a great central institution for medical relief and instruction. Besides the amount already collected, over six lakhs of rupees (about £40,000) were contributed on the spot.

The General Committee of the Leeds Workpeople's Hospital Fund has approved of a scheme recommended by the Executive Committee for the improving of the water supply at the Springfield Convalescent Home at Horsforth by building a reservoir to take in the whole of the water supply at the springs, and thereby to obtain a more adequate and constant supply of water for the Home. The scheme has also the additional advantage of providing a constant supply of water in case of fire. The cost will be £500.

The Government Hospital for the treatment of tuberculosis, at Fort Stanton, New Mexico, may have to be abandoned because of the failure of its water supply. Its water supply, hitherto abundant, has, it is said, been virtually cut off by the action of a railroad which is piping the water to a point thirty miles beyond for use in locomotives. So unless some means of supplying water is found, the institution, which has cost nearly a million dollars, will have to go out of commission.

The minute of the sub-committee of the Eastern District of Stirling County Council that the extension of the East Stirlingshire Fever Hospital be proceeded with next spring at a cost of £1,500 has been adopted. Dr. McVail, the medical officer, said that time should be taken to decide on the respective merits of the various types of diphtheria wards recently advocated.

The Holt Children's Sanatorium, Norfolk, has issued an appeal for £6,000 to double its accommodation. It is one of the few sanatoria for phthisical children in England, and its effects have been so good that the present accommodation for twenty children does not supply the demand. For further particulars readers are referred to The Hospital for June 11, page 333. Subscriptions should be sent to Mr. T. H. Wyatt, M.V.O., at the London offices, 68 Denison House, Vauxhall Bridge Road.

### TO HOSPITAL SECRETARIES AND OTHERS.

We invite contributions from any of our readers, and shall be glad to pay, according to length, for items of news for the institutional section (including appointments, resignations, gifts, etc.) which we may publish. All payments for manuscripts used are made as early as possible after the beginning of each quarter.

### THE HOSPITAL WORLD.

BY a simple but memorable ceremony, the beautiful little cottage hospital of St. Monica, Easingwold, was last week formally handed over by the founder (Mrs. Love) to a committee representing the town and district, in the presence of an influential gathering of subscribers and others.

ON September 12 the Postmaster-General will visit Benenden Sanatorium for the purpose of formally handing over to the Association which owns the sanatorium a pavilion of twenty beds which has been erected and equipped by Post Office subscribers. The total cost of this building has not exceeded £900.

AT the meeting of the Gas Light and Coke Company, on the 5th inst., it was foreshadowed that there would be a further reduction in the price of gas at the end of this year, making the seventh reduction in eight years. This continuous decrease in the price charged by the Gas Light Company is very welcome to the consumers, who benefit to the extent of nearly £100,000 a year by every reduction of 1d. per 1,000 cubic feet.

The sub-committee of the Johannesburg Hospital Board has reported on the recent confusion at the hospital mortuary—as a result of which the remains of one lady were interred as those of another. The report finds that there was carelessness, and severely reprimands the mortuary attendant.

THE HOSPITAL. August 20, 1910.

*Name* ..................................

*Address* ..................................

..................................

This Coupon must accompany manuscript or contributions intended for The Hospital.

